# Wheat Bax Inhibitor-1 interacts with TaFKBP62 and mediates response to heat stress

**DOI:** 10.1186/s12870-018-1485-0

**Published:** 2018-10-26

**Authors:** Pan-Pan Lu, Wei-Jun Zheng, Chang-Tao Wang, Wen-Yan Shi, Jin-Dong Fu, Ming Chen, Jun Chen, Yong-Bin Zhou, Ya-Jun Xi, Zhao-Shi Xu

**Affiliations:** 1 Key Laboratory of Biology and Genetic Improvement of Triticeae Crops, Ministry of Agriculture, Institute of Crop Science, Chinese Academy of Agricultural Sciences (CAAS)/National Key Facility for Crop Gene Resources and Genetic Improvement, Beijing, 100081 China; 2 Beijing Key Lab of Plant Resource Research and Development, Beijing Technology and Business University, Beijing, 100048 China; 3 College of Agronomy, Northwest A&F University, Yangling, 712100 Shaanxi China

**Keywords:** Bax inhibitor-1, Heat stress, TaFKBP62, RNA-seq, Heat-responsive genes

## Abstract

**Background:**

Heat stress is a severe environmental stress that affects plant growth and reduces yield. Bax inhibitor-1 (BI-1) is a cytoprotective protein that is involved in the response to biotic and abiotic stresses. The *Arabidopsis* (*Arabidopsis thaliana*) BI-1 mutants *atbi1–1* and *atbi1–2* are hypersensitive to heat stress, and *AtBI-1* overexpression rescues thermotolerance deficiency in *atbi1* plants. Nevertheless, the mechanism of BI-1 in plant thermotolerance is still unclear.

**Results:**

We identified a wheat (*Triticum aestivum* L.) BI-1 gene, *TaBI-1.1*, which was highly upregulated in an RNA sequencing (RNA-seq) analysis of heat-treated wheat. The upregulation of *TaBI-1.1* under heat stress was further demonstrated by real time quantitative PCR (qRT-PCR) and β-glucuronidase (GUS) staining. Compared with the wild type Col-0, the *atbi1–2* mutant is hypersensitive to heat stress, and constitutive expression of *TaBI-1.1* in *atbi1–2* (*35S::TaBI-1.1/ atbi1–2*) rescued the deficiency of *atbi1–2* under heat stress. Furthermore, we identified TaFKBP62 as a TaBI-1.1-interacting protein that co-localized with TaBI-1.1 on the endoplasmic reticulum (ER) membrane and enhanced heat stress tolerance. Additionally, *HSFA2*, *HSFB1*, *ROF1*, *HSP17.4B*, *HSP17.6A*, *HSP17.8*, *HSP70B*, and *HSP90.1* expression levels were suppressed in *atbi1–2* plants under heat stress. In contrast, *35S::TaBI-1.1/atbi1–2* relieved the inhibitory effect of *AtBI-1* loss of function.

**Conclusions:**

TaBI-1.1 interacted with TaFKBP62 and co-localized with TaFKBP62 on the ER membrane. Both TaBI-1.1 and AtBI-1 regulated the expression of heat-responsive genes and were conserved in plant thermotolerance.

**Electronic supplementary material:**

The online version of this article (10.1186/s12870-018-1485-0) contains supplementary material, which is available to authorized users.

## Background

Heat stress is a major environmental factor that affects almost all aspects of plant growth, development, reproduction, and yield. High temperature affects the stability of multiple proteins and cytoskeletal structures and alters the efficiency of enzymatic reactions, and exposure to sustained high temperature can even result in plant death [1].

Plants have evolved a variety of mechanisms to resist heat stress, including the maintenance of membrane stability, ROS scavenging, antioxidant production, the accumulation and adjustment of compatible solutes, the induction of protein kinase cascades, chaperone signalling, and transcriptional activation [2]. Heat shock proteins (HSPs) are important components of the response to heat stress that can improve photosynthesis, assimilate partitioning, water and nutrient use efficiency, and membrane stability [3–5]. HSPs are induced through the action of heat-stress transcription factors (HSFs), which mediate the synthesis of HSPs by binding to heat-stress elements in the promoters of heat-responsive genes.

Bax inhibitor-1 (BI-1) is a cell death suppressor that is conserved in plants and mammals [6, 7]. BI-1 was first identified in mammals but was functionally verified in yeast and shown to suppress Bax-induced cell death [8]. Programmed cell death (PCD) is a conserved process in eukaryotes induced by stress stimuli or during development [7]. Numerous studies have revealed that BI-1 inhibits cell death induced by biotic and abiotic stresses. Overexpressing *Arabidopsis* (*Arabidopsis thaliana*) *AtBI-1* in tobacco BY-2 cells suppresses Bax-, H_2_O_2_- or salicylic acid (SA)-mediated cell death [9]. Overexpressing *TaBI-1* in tobacco blocks Bax-induced cell death, and silencing *TaBI-1* in wheat (*Triticum aestivum* L.) enhances the susceptibility to *Puccinia striiformis* [10]*. HvBI-1* overexpression increases susceptibility to biotrophic *Blumeria graminis* f.sp. *hordei* but enhances resistance to *Fusarium graminearum* (*Fg*) and inhibits cell death induced by mammalian BAX expression [11]. Enhanced barley *BI-1* expression suppresses penetration resistance to *B. graminis* f. sp. *tritici* [12]. *AtBI-1* overexpression in sugarcane increases tolerance to long-term water deficit [13]. Recent studies suggest that BI-1 is involved in the response to heat stress. MrBI-1 partially rescues Bax-induced cell death in yeast, and deleting *MrBI-1* impairs heat tolerance [14]. Pepper *CaBI-1* is upregulated by high temperature [15]. Two *AtBI-1* mutants (*atbi1–1* and *atbi1–2*) exhibit hypersensitivity to tunicamycin (TM)-induced PCD progression and increased sensitivity to heat shock-induced cell death. In contrast, *AtBI-1* overexpression rescues thermotolerance deficiency in *atbi1* plant [16, 17]. However, the mechanism of BI-1 in thermotolerance is unclear.

FK506-binding proteins (FKBPs) are a superfamily of peptidyl prolyl cis-trans isomerases (PPIases) that are characterized by their enzymatic activity [18, 19]. The catalytic activity of FKBP is inhibited upon binding of the immunosuppressive drug FK506 [20]. FKBP family members are found in multiple subcellular locations, including the endoplasmic reticulum (ER) [21], cytosol [22], nucleus [23], and mitochondria [24]. FKBP isoforms are distinguished by their molecular weights, which range from 12 kDa to over 77 kDa [25–27]. Large FKBPs contain additional domains that enable them to function as chaperones, such as AtFKBP62 (ROF1) and AtFKBP65 (ROF2). ROF1 and ROF2 share similar domains and high sequence identity. These proteins contain three peptidylprolyl cis/trans isomerases (PPIase) domains, a tetratricopeptide repeat motif (TPR) domain, and a calmodulin-binding domain [28]. The first PPIase domain possesses PPIase activity and an FK506 binding site, while the remaining two PPIase-like domains exhibit only partial identity to the functional domain [27]. The TPR domain is necessary for ROF1 to interact with HSP90 [29]. The expression levels of both ROF1 and ROF2 increase under heat stress, but ROF2 is detected only after heat treatment [29]. ROF1 plays a role in prolonging thermotolerance by sustaining the levels of the small HSPs that are necessary under heat stress [30]. Nevertheless, the functions of large FKBPs in other species are largely unknown.

During the late growth stage of wheat, dry hot winds can substantially reduce grain filling. Currently, no studies have investigated the involvement of wheat BI-1 in the response to heat stress, and little is known about the function of AtBI-1 in plant thermotolerance. In our previous work, we identified the ER-resident protein TaBI-1.1 from an RNA sequencing (RNA-seq) analysis of *Fg*-treated wheat. Here, we demonstrated that TaBI-1.1, a TaFKBP62-interacting protein, is conserved with AtBI-1 in the response to heat stress and that both TaBI-1.1 and AtBI-1 positively regulate heat-responsive genes expression.

## Results

### TaBI-1.1 is upregulated under heat stress

**Fig. 1:**
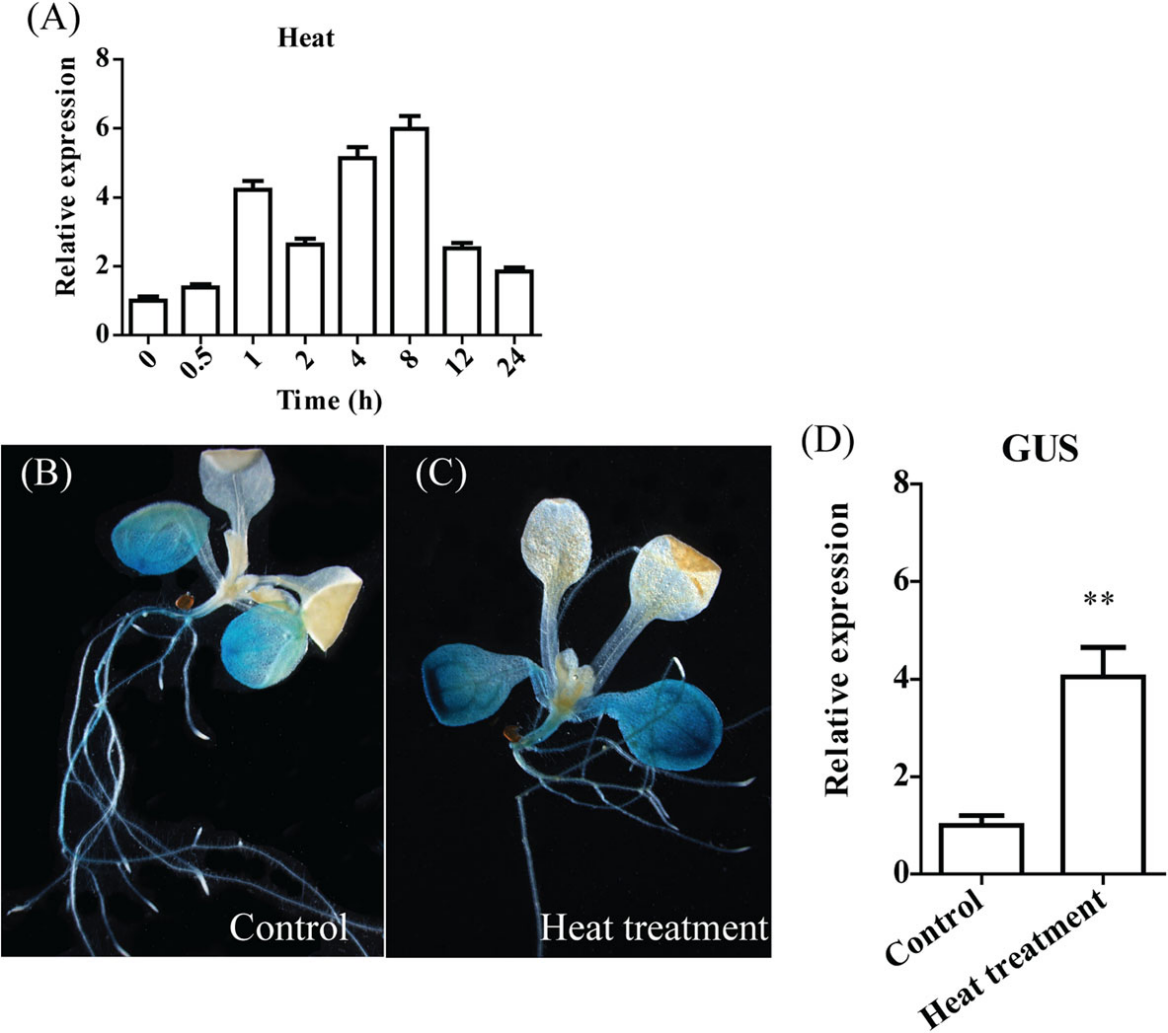
Relative and spatial expression patterns of *TaBI-1.1*. **a** Expression profile of *TaBI-1.1* under heat treatment for 0, 0.5, 1, 2, 4, 8, 12, and 24 h. Wheat *Actin* was used as a reference. The vertical coordinates are the fold-changes, and the horizontal ordinates are the different time periods. **b** and **c** GUS activity of PBI::GUS transgenic *Arabidopsis* under normal conditions (**b**) and heat treatment (**c**) was detected via GUS histochemical staining. Seedlings grown under normal conditions were used as controls. **d** GUS expression levels in the PBI:GUS transgenic lines were monitored by qRT-PCR. *Actin2* was used as a reference. The results are shown as the means±standard deviation (SD) of three biological replicates. Error bars indicate the SD. Asterisks (**) indicate the significant differences (*P* < 0.01) compared with Col-0 (Student’s t-test)

We analysed the RNA-seq data from heat-treated wheat to investigate the biological mechanism of the heat stress response. A highly upregulated gene, *TaBI-1.1* (12.15-fold increase, TRIAE_CS42_U_TGACv1_644608_AA2140670), was identified from the RNA-seq data (Additional file 1: Table S1). To determine whether *TaBI-1.1* was upregulated under heat stress, we monitored *TaBI-1.1* expression using real time quantitative PCR (qRT-PCR). *TaBI-1.1* mRNA accumulated during heat treatment, reaching a peak of ~ 6-fold at 8 h (Fig. 1a). We analysed the histological β-glucuronidase (GUS) activity via GUS staining of PBI::GUS transgenic *Arabidopsis* to investigate the spatial expression pattern of *TaBI-1.1*. Higher levels of GUS protein accumulation were observed in the mature leaves of heat-treated plants than in those of control plants (Fig. 1b and c). GUS mRNA levels were also obviously increased, as shown by qRT-PCR, confirming the upregulation of *TaBI-1.1* under heat stress (Fig. 1d).

### Constitutive expression of *TaBI-1.1* in *atbi1–2* fully rescues defective heat tolerance in *atbi1–2* plants

**Fig. 2:**
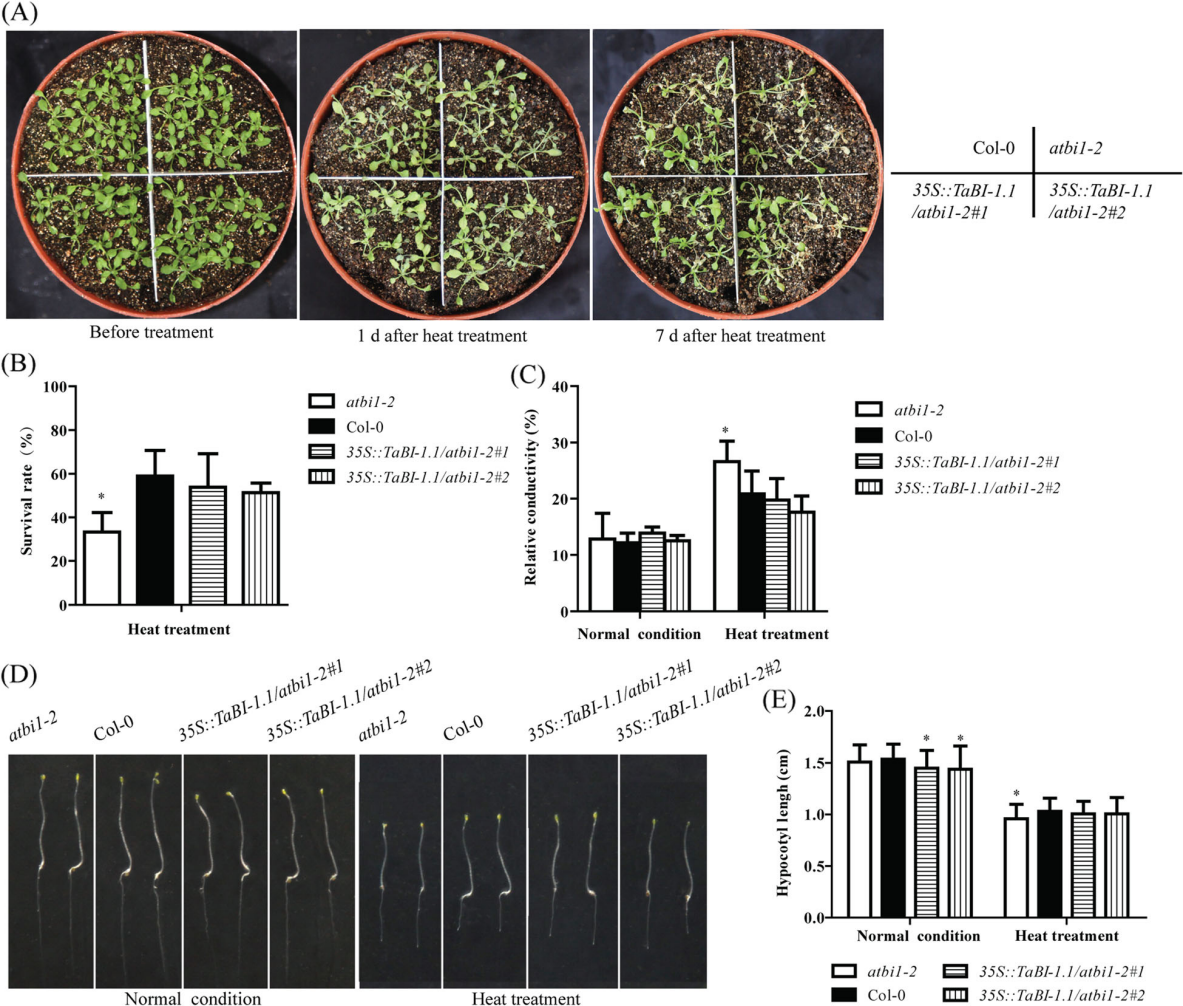
Constitutive *TaBI-1.1* expression in *atbi1–2* rescues the deficiency of *atbi1–2* in heat tolerance. **a** The phenotypes of *atbi1–2*, Col-0, *35S::TaBI-1.1/atbi1–2#1*, and *35S::TaBI-1.1/atbi1–2#2* under heat stress. **b** The survival rates of *atbi1–2*, Col-0, *35S::TaBI-1.1/atbi1–2#1*, and *35S::TaBI-1.1/atbi1–2#2* under heat stress. The results are shown as the means±SD of three biological replicates. Error bars indicate the SD. **c** The relative conductivity of *atbi1–2*, Col-0, *35S::TaBI-1.1/atbi1–2#1*, and *35S::TaBI-1.1/atbi1–2#2* after heat treatment. The results are shown as the means±SD of six biological replicates. Error bars indicate the SD. **d** Hypocotyl elongation in *atbi1–2*, Col-0, *35S::TaBI-1.1/atbi1–2#1*, and *35S::TaBI-1.1/atbi1–2#2* after heat treatment. **e** Statistical analysis of hypocotyl elongation in *atbi1–2*, Col-0, *35S::TaBI-1.1/atbi1–2#1*, and *35S::TaBI-1.1/atbi1–2#2* after heat treatment. The results are shown as the means±SD of 36 biological replicates. Error bars indicate the SD. All the asterisks in the figure (* and **) indicate significant differences (*P* < 0.05 and *P* < 0.01, respectively) compared with Col-0 (Student’s t-test)

BI-1 is known to be highly evolutionarily conserved. *atbi1–2* mutants are hypersensitive to heat stress, and *AtBI-1* overexpression can rescue this deficiency [17]. Based on the high upregulation of *TaBI-1.1* under heat treatment, we surmised that TaBI-1.1 might play a similar role as AtBI-1 in the response to heat stress. To test this hypothesis, we generated transgenic lines that ectopically expressed TaBI-1.1 in the *atbi1–2* background under the control of the cauliflower mosaic virus (CaMV) 35S promoter. Two homozygous lines with relatively high *TaBI-1.1* expression levels (*35S::TaBI-1.1/atbi1–2#1* and *35S::TaBI-1.1/atbi1–2#2*) were selected for further analysis. Eighteen-day-old *atbi1–2* and Col-0 plants, as well as the two transgenic lines, were exposed to 45 °C for 6 h, and the survival rates were tested after 7 days. The survival rates of Col-0, *35S::TaBI-1.1/atbi1–2#1*, and *35S::TaBI-1.1/atbi1–2#2* plants were not significantly different, and all the three genotypes exhibited significantly higher survival rates than *atbi1–2*. Compared with the survival rates of *atbi1–2* and Col-0, the two transgenic lines fully rescued the deficiency of *atbi1–2* under heat stress (Fig. 2a and b). Furthermore, we examined ion leakage in the different genotypes. Under normal conditions, no differences in relative conductivity were observed between any of the genotypes. However, under heat treatment, *atbi1–2* showed a significantly higher relative conductivity than Col-0 and the two transgenic lines, and no differences in relative conductivity were detected between Col-0 and the two transgenic lines, which confirmed that TaBI-1.1 fully rescued the deficiency of *atbi1–2* in response to heat stress, indicating the conserved function of BI-1 between wheat and *Arabidopsis* (Fig. 2c).

Hypocotyl elongation is known to be inhibited by heat stress [31]. To elucidate the role of BI-1 during hypocotyl elongation under heat stress, plants from all four genotypes were grown in the dark for 3 days and then exposed to 45 °C for 2 h. Hypocotyl elongation was tested after 3 days of recovery. Six-day-old plants grown in the dark were used as the control. In the control group, no differences in hypocotyl elongation were observed between *atbi1–2* and Col-0, while the two transgenic lines displayed significantly shorter hypocotyls than Col-0. In contrast, *atbi1–2* exhibited less hypocotyl elongation than the two transgenic lines and Col-0 under heat treatment (Fig. 2d and e). These results suggested that hypocotyl elongation in *atbi1–2* is hypersensitive under heat stress and that TaBI-1.1 rescues the hypersensitive phenotype of *atbi1–2*.

Based on the conserved function of TaBI-1.1 and AtBI-1 under heat stress, as well as their high sequence conservation, we analysed the number of BI-1 members in some important species by screening the Ensembl plant database and constructed a phylogenetic tree of the BI-1 family (Additional file 2: Figure S1B). *Aegilops tauschii*, *Arabidopsis thaliana*, *Brachypodium distachyon*, *Hordeum vulgare*, and *Oryza sativa* each contained only one BI-1. Additionally, only three members were identified in *Triticum aestivum* and *Zea mays* (Additional file 2: Figure S1A). These results showed that several BI-1 members are present in plant species, revealing the pivotal role of BI-1 in plants.

### TaBI-1.1 interacts with the TPR domain of TaFKBP62

To further explore the cellular mechanisms of TaBI-1.1 in heat tolerance, we performed yeast two-hybrid assays using TaBI-1.1 expressed from the pGBKT7 (BD) vector as the bait protein to screen a wheat cDNA library. One candidate interacting partner, the ROF1 homologue TaFKBP62, which shares 59.23% amino acid sequence identity with ROF1, was obtained. Both proteins contain three PPIase domains and one TPR domain and belong to the FKBP62 family. Their TPR domains share 70.94% identity, indicating that the TPR domain of FKBP62 is conserved between wheat and *Arabidopsis*.

**Fig. 3:**
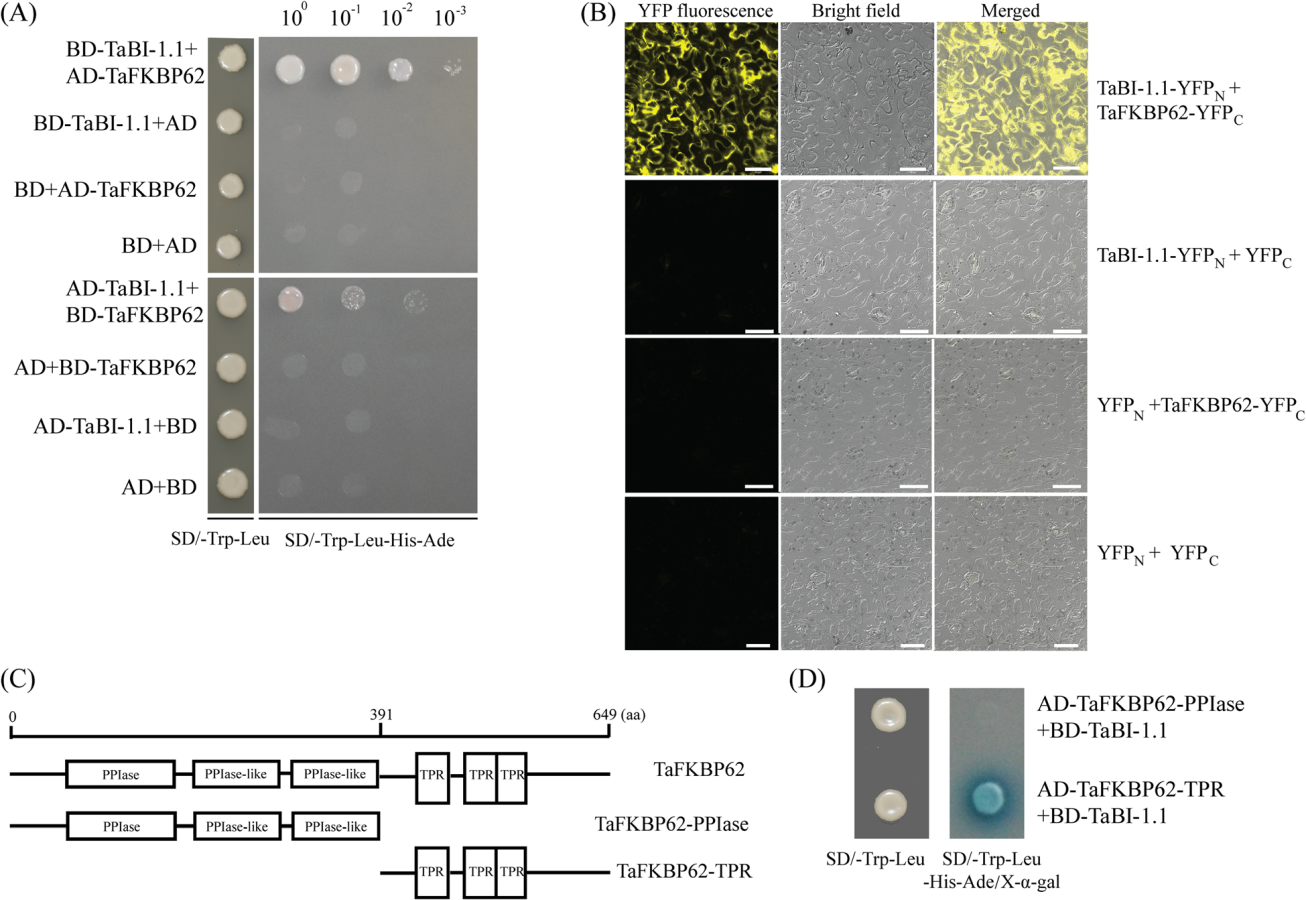
The interaction between TaBI-1.1 and TaFKBP62. **a** The interaction between TaBI-1.1 and TaFKBP62 measured using yeast two-hybrid assays. **b** The interaction between TaBI-1.1 and TaFKBP62 measured via BiFC assays in *Nicotiana benthamiana* epidermal cells. Bars = 50 μm. **c** The two fragments, TaFKBP62-PPIase and TaFKBP62-TPR, corresponding to the PPIase and TPR domain, respectively, of TaFKBP62. **d** Interactions between TaBI-1.1 and the two TaFKBP62 fragments detected via yeast two-hybrid assay

Yeast two-hybrid and bimolecular fluorescence complementation (BiFC) assays were used to explore the interaction between TaBI-1.1 and TaFKBP62. A BD vector containing TaBI-1.1 (BD-TaBI-1.1) and a pGADT7 (AD) vector containing TaFKBP62 (AD-TaFKBP62) were constructed for the yeast two-hybrid analysis. Four groups, BD-TaBI-1.1 + AD-TaFKBP62, BD-TaBI-1.1 + AD, BD + AD-TaFKBP62, and BD + AD, were co-transformed into yeast cells. Only yeast cells transformed with BD-TaBI-1.1 and AD-TaFKBP62 were able to grow on selective medium lacking Trp, Leu, His, and Ade (SD/−Trp-Leu-Ade-His). Conversely, co-transformants expressing BD-TaBI-1.1 + AD, BD + AD-TaFKBP62 or BD + AD did not grow on SD/−Trp-Leu-Ade-His medium. Similar results were obtained when we switched the TaBI-1.1 and TaFKBP62 vectors, indicating that TaBI-1.1 interacted with TaFKBP62 in yeast cells (Fig. 3a). This interaction was further demonstrated using BiFC assay. Four vector groups, TaBI-1.1–YFP_N_ (N-terminal fragment of yellow fluorescent protein) and TaFKBP62–YFP_C_ (C-terminal fragment of yellow fluorescent protein), TaBI-1.1–YFP_N_ and YFP_C_, YFP_N_ and TaFKBP62–YFP_C_, and YFP_N_ and YFP_C_, were injected into *Nicotiana benthamiana* leaves. YFP fluorescence was only detected in leaf epidermal cells injected with TaBI-1.1–YFP_N_ and TaFKBP62–YFP_C_ (Fig. 3b), confirming the interaction between TaBI-1.1 and TaFKBP62.

To determine which region of TaFKBP62 interacted with TaBI-1.1, the TaFKBP62 sequence was divided to two fragments, TaFKBP62-PPIase (N-terminal and three PPIase domains) and TaFKBP62-TPR (TPR domain), according to the protein domains (Fig. 3c). The two fragments were cloned into an AD vector and co-transformed with BD-TaBI-1.1 into yeast cells. Both AD-TaFKBP62-PPIase + BD-TaBI-1.1 and AD-TaFKBP62-TPR + BD-TaBI-1.1 cells grew on SD/−Trp-Leu medium, whereas only transformants expressing AD-TaFKBP62-TPR and BD-TaBI-1.1 grew on SD/−Trp-Leu-His-Ade/X-α-gal medium (Fig. 3d). These results suggested that TaBI-1.1 specifically interacts with the TPR domain of TaFKBP62.

Given the conserved TPR domain between TaFKBP62 and ROF1, the sequence conservation between TaBI-1.1 and AtBI-1, and the interaction between TaFKBP62 and TaBI-1.1, we also tested the interaction between AtBI-1 and ROF1, as well as AtBI-1 and the TPR domain of ROF1. However, AtBI-1 did not interact with ROF1 or the TPR domain of ROF1 in yeast cells (Additional file 3: Figure S2).

### TaFKBP62 co-localizes with TaBI-1.1 on the ER membrane and enhances the heat stress tolerance in *Arabidopsis*

**Fig. 4:**
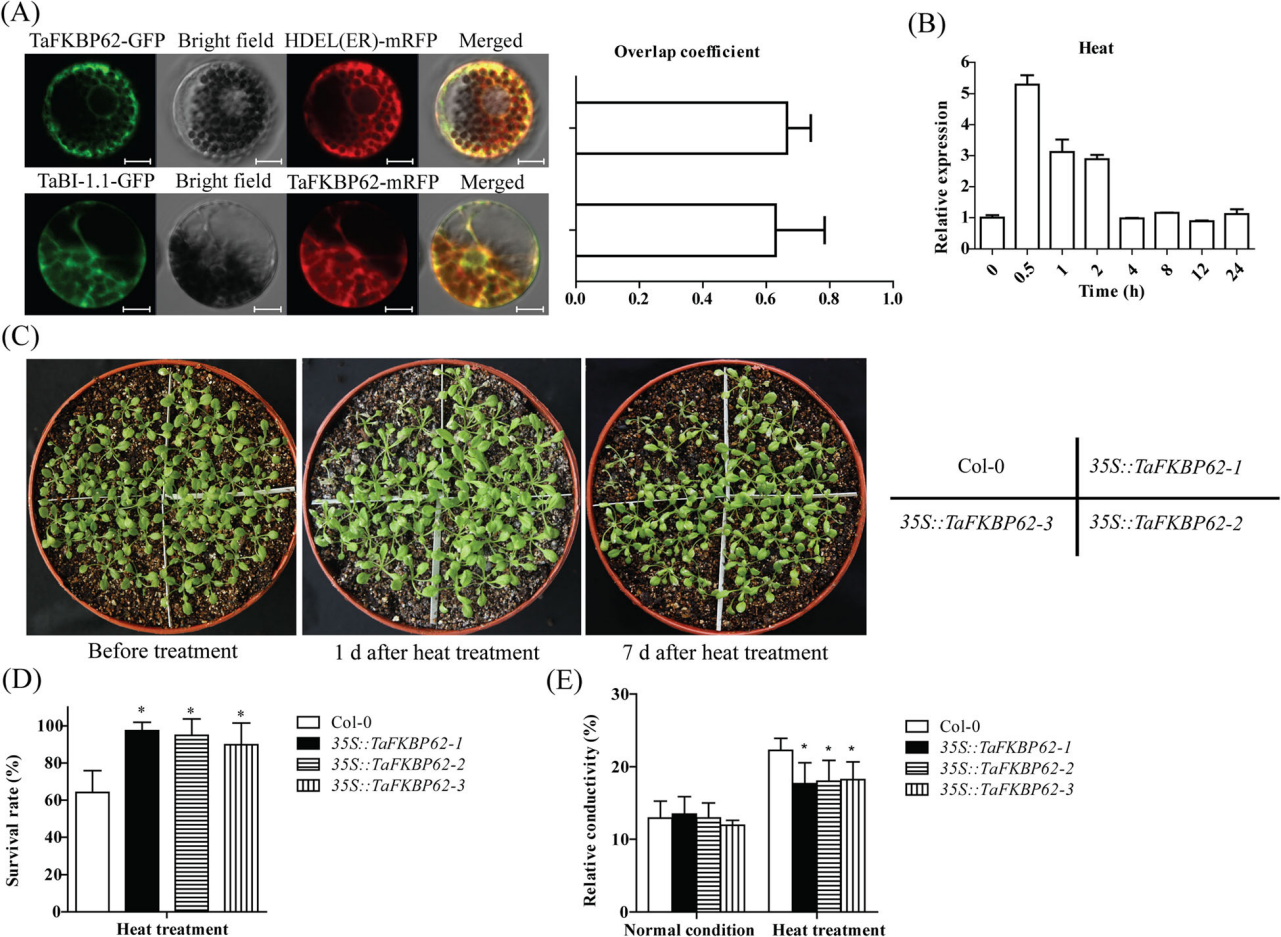
The co-localization between TaBI-1.1 and TaFKBP62 and *TaFKBP62* expression patterns under stress treatments. **a** The subcellular localization of TaFKBP62 and the co-localization between TaBI-1.1 and TaFKBP62 in wheat protoplasts. The left panel shows the fluorescence of GFP, mRFP and the merge. Bars = 10 μm. The right panel shows the co-localization levels calculated from the overlap coefficients obtained from at least ten individual protoplasts. **b** *TaFKBP62* expression patterns after heat treatment for 0, 0.5, 1, 2, 4, 8, 12, and 24 h. Wheat *Actin* was used as a reference. The vertical coordinates represent the fold-changes, and the horizontal ordinates represent the different time periods. The results are shown as the means±SD of three biological replicates. Error bars indicate the SD. **c** The phenotypes of Col-0, *35S::TaFKBP62–1*, *35S::TaFKBP62–2,* and *35S::TaFKBP62–3* after heat stress. **d** The survival rates of Col-0, *35S::TaFKBP62–1*, *35S::TaFKBP62–2,* and *35S::TaFKBP62–3* under heat stress. The results are shown as the means±SD of three biological replicates. Error bars indicate the SD. **e** The relative conductivity of Col-0, *35S::TaFKBP62–1*, *35S::TaFKBP62–2,* and *35S::TaFKBP62–3* after heat treatment. The results are shown as the means±SD of six biological replicates. Error bars indicate the SD

We previously showed that TaBI-1.1 localizes to the ER membrane [32]. In view of the interaction between TaBI-1.1 and TaFKBP62, we constructed recombinant TaFKBP62-GFP and TaFKBP62-mRFP vectors to detect the subcellular localization of TaFKBP62 and to determine whether TaBI-1.1 co-localizes with TaFKBP62 at the ER membrane. Two groups, TaFKBP62-GFP + mRFP-HDEL (an ER marker) and TaBI-1.1-GFP + TaFKBP62-mRFP, were co-transformed into wheat protoplasts. The overlap coefficient for TaFKBP62-GFP and mRFP-HDEL fluorescence was 0.67, indicating that TaFKBP62 localized to the ER membrane in the wheat protoplasts (Fig. 4a). The overlap coefficient for TaBI-1.1-GFP and TaFKBP62-mRFP fluorescence was 0.63, suggesting that TaBI-1.1 co-localized with TaFKBP62 at the ER membrane (Fig. 4a). ROF1 localizes to the cytoplasm under normal conditions and translocates into the nucleus under heat treatment [30]. However, we did not observe the nuclear translocation of either TaFKBP62 or TaBI-1.1 under heat stress (Additional file 4: Figure S3).

To determine the possible role of TaFKBP62 under heat stress, we monitored *TaFKBP62* expression patterns using qRT-PCR and found that *TaFKBP62* obviously accumulated during heat treatment, reaching a peak point of ~ 5.3-fold at 0.5 h (Fig. 4b). *TaFKBP62* upregulation was also detected in the RNA-seq data from heat-treated wheat (Additional file 5: Table S2). We generated transgenic *Arabidopsis* plants that constitutively expressed TaFKBP62 under the control of the CaMV 35S promoter. Three homozygous lines, *35S::TaFKBP62–1 35S::TaFKBP62–2* and *35S::TaFKBP62–3*, were selected for further analysis. Eighteen-day-old Col-0 and the three transgenic lines were exposed to 45 °C for 6 h, and survival rates were tested after 7 days. The survival rates of the three transgenic lines were significantly higher than those of Col-0 (Fig. 4c and d). Ion-leakage assays showed that the three transgenic lines exhibited significantly lower relative conductivity than Col-0 under heat stress, indicating that *TaFKBP62* enhanced heat stress tolerance in *Arabidopsis* (Fig. 4e).

### TaBI-1.1 is conserved with AtBI-1 in regulating heat-responsive gene expression

**Fig. 5:**
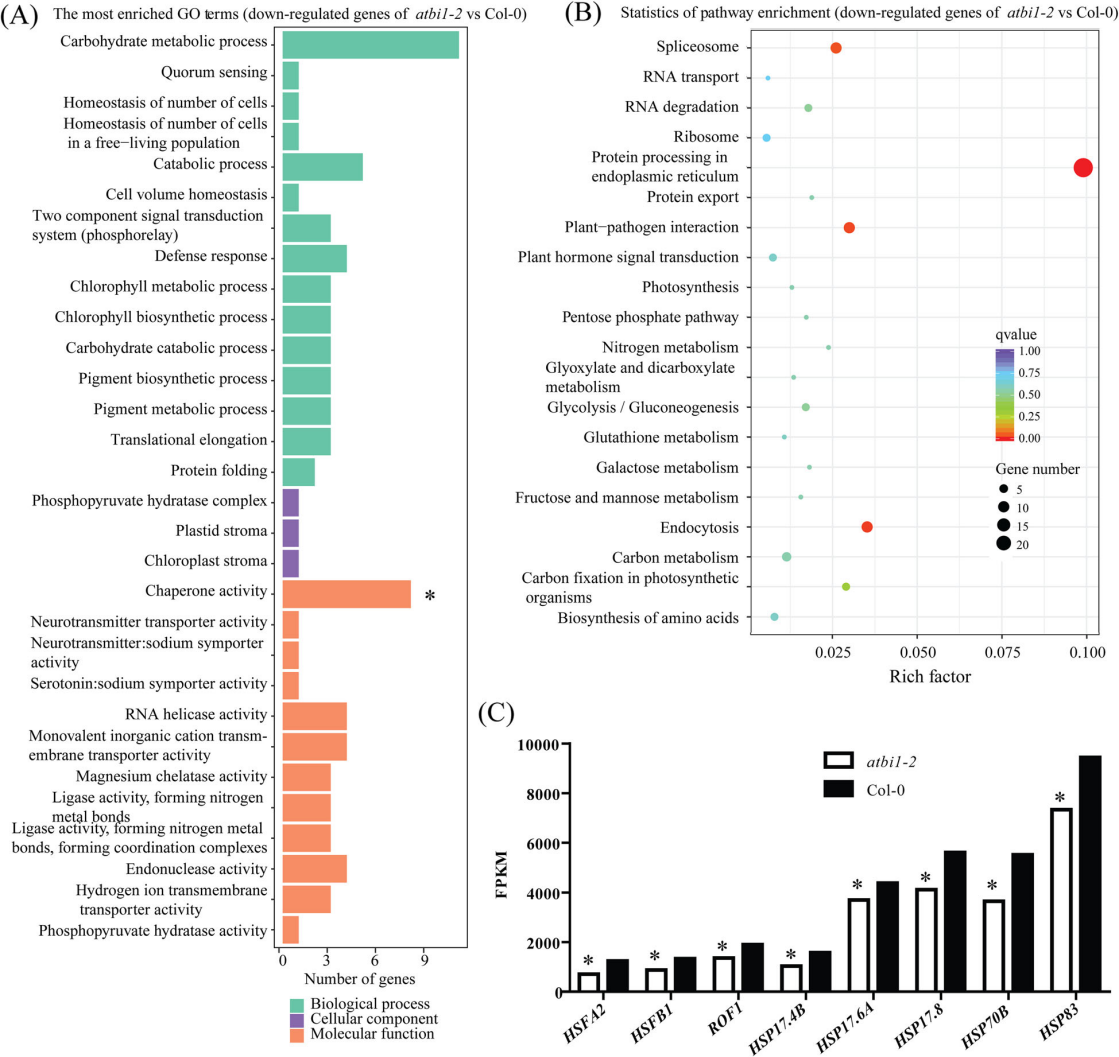
GO term enrichment and KEGG enrichment analyses were conducted using the RNA-seq data. **a** GO term enrichment analysis of the downregulated genes. The vertical coordinates are the enriched GO terms, and the horizontal coordinates are the numbers of the downregulated genes in these GO terms. The green columns represent the biological process GO terms; the purple columns represent the cellular component GO terms; the orange columns represent the molecular function GO terms. “*” indicates a significantly enriched GO term. **b** KEGG enrichment analysis of the downregulated genes. The vertical coordinates are the enriched pathways, and the horizontal coordinates are the rich factors. The size of each point represents the number of downregulated genes in the pathway, and the colour of the point represents the *q*-value. **c** FPKM values of the eight downregulated genes. Asterisks (*) indicate significant differences (padj< 0.005) compared with Col-0

We conducted an RNA-seq analysis of *atbi-1* and Col-0 under heat treatment to further investigate the mechanism of BI-1 in response to heat stress. Thirty-five upregulated genes and 80 downregulated genes were identified (Additional file 6: Figure S4). The gene identity (ID) numbers and fold-changes are shown in Additional file 5: Table S2. Most of the HSPs were positively regulated under heat stress. Therefore, we analysed the downregulated genes in *atbi-1* versus (vs) Col-0. The top 30 enriched Gene Ontology (GO) terms and the top 20 enriched Kyoto Encyclopaedia of Genes and Genomes (KEGG) pathways among the downregulated genes are shown in Fig. 5a and b. Only the term chaperone activity was significant among the 30 enriched GO terms. Of the top 20 enriched pathways, the greater enrichment factor represented a higher degree of enrichment. The enriched pathway protein processing in the ER had the highest enrichment factor, indicating that most of the downregulated genes were involved in the protein processing pathway in the ER. Eight differentially expressed genes, *HSFA2*, *HSFB1*, *ROF1*, *HSP17.4B*, *HSP17.6A*, *HSP17.8*, *HSP70B*, and *HSP90.1*, were selected to generate a histogram for visual analysis based on their fragments per kilobase of transcript per million mapped reads (FPKM) in *atbi-1* and Col-0. The FPKM of these genes was significantly lower in *atbi-1* than in Col-0 (Fig. 5c), revealing that *atbi1–2* deficiency might be caused by the downregulation of these genes under heat stress and that the loss of *AtBI-1* function might affect protein processing and chaperone activity under heat stress.

**Fig. 6:**
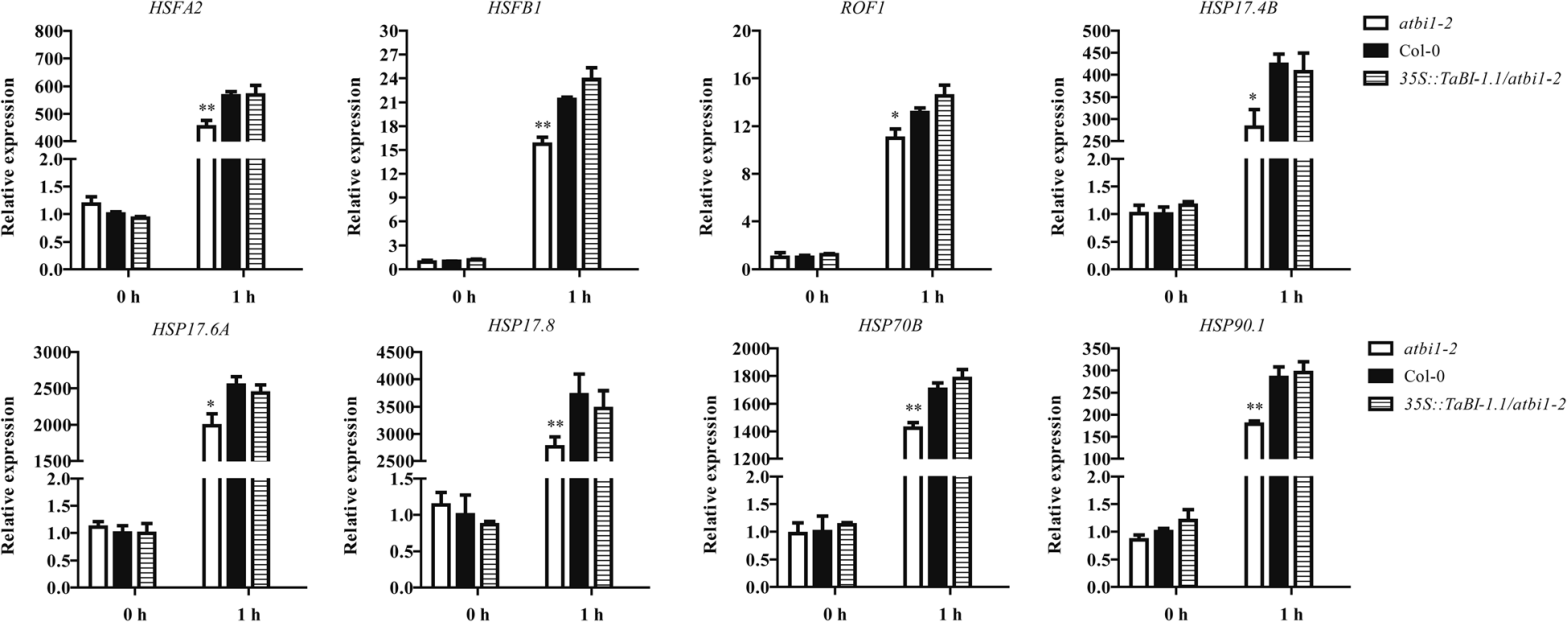
The expression levels of eight genes in *atbi1–2*, Col-0 and *35S::TaBI-1.1/atbi1–2* detected by qRT-PCR after heat treatment. Gene expression levels in Col-0 at 0 h were set to “1.” The results are shown as the means±SD of three biological replicates. Error bars indicate the SD. Asterisks (* and **) indicate significant differences (*P* < 0.05 and *P* < 0.01, respectively) compared with Col-0 (Student’s t-test)

To verify the accuracy of the RNA-seq analysis and investigate whether TaBI-1.1 can rescue the downregulation of these genes in *atbi1–2*, qRT-PCR analyses were performed using the aforementioned eight genes. Under normal conditions (0 h), no significant differences in gene expression levels were detected among the three genotypes. After heat treatment for 1 h, all eight genes showed obvious upregulation, indicating that they were heat-responsive genes. The expression levels of these genes were significantly lower in *atbi1–2* than in Col-0. In contrast, no significant differences in gene expression were observed between Col-0 and *35S::TaBI-1.1/atbi1–2* (Fig. 6). These results suggested that TaBI-1.1 fully rescued the downregulation of these heat-responsive genes in *atbi1–2*.

## Discussion

### TaBI-1.1 is conserved with AtBI-1 in response to heat stress

BI-1 is an ER-resident cell death suppressor that is highly evolutionarily conserved across species. Numerous studies have shown that plant BI-1 functions as an attenuator of cell death induced by biotic and abiotic stresses such as pathogens, heat, cold, drought, and salt. Human BI-1 blocks cell death induced by the ectopic expression of the mouse Bax [8]. The overexpression of rice and *Arabidopsis* BI-1 genes in yeast and plant cells also suppresses cell death induced by mammalian Bax [32, 33]. *Arabidopsis BI-1* is upregulated under heat stress [17]. The two mutants, *atbi1–1* and *atbi1–2*, exhibit increased sensitivity to heat stress, and *BI-1* overexpression rescues the deficiency of the mutants [17]. In the present study, we demonstrated that *TaBI-1.1* is a heat-responsive gene and that overexpressing *TaBI-1.1* in *atbi1–2* rescues the hypersensitive phenotype of *atbi1–2* under heat stress (Figs. 1 and 2). Eight heat-responsive genes, *HSFA2*, *HSFB1*, *ROF1*, *HSP17.4B*, *HSP17.6A*, *HSP17.8*, *HSP70B*, and *HSP90.1*, were significantly downregulated in *atbi1–2* compared with their levels in Col-0 under heat stress, whereas the downregulated expression levels were fully supplemented in *35S::TaBI-1.1/atbi1–2* (Figs. 5c and 6), indicating that TaBI-1.1 is conserved with AtBI-1 in response to heat stress.

The sequence of BI-1 is highly conserved in plants, and only a few BI-1 members were identified in each plant species (Additional file 2: Figure S1), revealing a crucial role of BI-1 in plants. In view of the conserved function of BI-1 between wheat and *Arabidopsis* in response to heat stress, we surmised that BI-1 might also be involved in thermotolerance in other plant species.

### The interaction between TaBI-1.1 and TaFKBP62 may be involved in heat tolerance

TPR-containing proteins exhibit a large degree of sequence diversity, but structural comparison reveals three conserved helical coiled structures formed by the concatenation of ~ 34-residue helical hairpins [34]. Most identified HSP90 co-chaperones contain a TPR domain that binds to the MEEVD motif at the C-terminus of HSP90 [35, 36]. Human TPR-containing immunophilins FKBP52 and FKBP38 bind HSP90 through their TPR domains [37, 38]. *Arabidopsis* ROF1 binds HSP90.1 via its TPR domain [29]. The ROF1–HSP90.1 complex translocates into the nucleus and interacts with HSFA2 upon exposure to heat stress. However, HSFA2 only interacts with HSP90.1, but not with ROF1 [30]. Therefore, ROF1 mediates the interaction between HSP90.1 and HSFA2 through its TPR domain in response to heat stress [30]. In our study, TaBI-1.1 interacted with the TPR domain of TaFKBP62, and the TPR domain of FKBP62 was conserved between wheat and *Arabidopsis* (Fig. 3). Overexpressing *TaBI-1.1* in *atbi1–2* upregulated *ROF1* expression under heat treatment, indicating that TaBI-1.1 positively regulates *ROF1* expression under heat stress (Fig. 6). Constitutive TaFKBP62 expression increased tolerance to heat stress in *Arabidopsis* (Fig. 4). Thus, we hypothesized that the interaction between TaBI-1.1 and TaFKBP62 might be involved in thermotolerance.

Despite the high degree of sequence conservation between TaBI-1.1 and AtBI-1 and the conserved TPR domain between TaFKBP62 and ROF1, neither ROF1 nor the TPR domain of ROF1 interacted with AtBI-1 (Additional file 3: Figure S2). The ROF1–HSP90.1 complex localizes to the cytoplasm under normal conditions and translocates to the nucleus following exposure to heat stress [30]. In the present study, TaFKBP62 was found to be an ER-resident protein (Fig. 4a) and did not translocate to the nucleus under heat stress (Additional file 4: Figure S3), implying that the mechanisms of TaFKBP62 and ROF1 in plant thermotolerance were different. We hypothesized that TaFKBP62 may act as a TaBI-1.1 chaperonin through its TPR domain on the ER membrane in response to heat stress.

### BI-1 might respond to heat stress by regulating heat-responsive gene expression

HSFA2 acts as a positive regulator in the response to heat stress [39, 40]. HSFB proteins were suggested to be coactivators of HSFAs [41]. HSPs function in protein folding, the maintenance of protein stability, and the activation and maturation of cellular proteins through cooperation with client proteins and co-chaperones to regulate cellular processes [42–44]. Both TaBI-1.1 and AtBI-1 regulated the expression of *HSFA2*, *HSFB1*, *ROF1*, *HSP17.4B*, *HSP17.6A*, *HSP17.8*, *HSP70B*, and *HSP90.1* under heat stress (Figs. 5c and 6). Therefore, we hypothesized that the involvement of BI-1 in thermotolerance might be related to the regulation of heat-responsive genes.

In the RNA-seq analysis of heat-treated *atbi-1* and Col-0 plants, the most enriched GO term and pathway were chaperone activity and protein processing in the ER, respectively (Fig. 5a and b). AtBI-1, as an ER-resident protein, modulates ER stress-mediated PCD [7]. The loss of AtBI-1 function in *atbi1–2* negatively regulated *HSFA2*, *HSFB1*, *ROF1*, *HSP17.4B*, *HSP17.6A*, *HSP17.8*, *HSP70B*, and *HSP90.1* expression (Figs. 5c and 6). The ROF1–HSP90.1 complex interacts with HSFA2 upon exposure to heat stress to regulate the accumulation of small HSPs [30]. Therefore, we hypothesized that AtBI-1 regulates ROF1 and HSP90.1 protein processing at the ER membrane to affect the stability of the ROF1–HSP90.1–HSFA2 complex, resulting in the accumulation of small HSPs following exposure to heat stress.

## Conclusions

Compared with Col-0, *atbi1–2* mutants are hypersensitive to heat stress, and constitutive *TaBI-1.1* expression in *atbi1–2* plants rescued the deficiency of *atbi1–2* under heat stress. TaBI-1.1 interacted with the TPR domain of TaFKBP62. TaFKBP62 was found to be an ER-resident protein that co-localized with TaBI-1.1. Additionally, both TaBI-1.1 and AtBI-1 regulated the expression of *ROF1* in response to heat stress. Because constitutive *TaFKBP62* expression enhanced heat-stress tolerance in *Arabidopsis*, we hypothesized that the interaction between TaFKBP62 and TaBI-1.1 may be involved in the response to heat stress. Our results revealed the role of TaBI-1.1 and AtBI-1 in response to heat stress and the possible relationship between BI-1 and FKBP62 in *Arabidopsis* and wheat. Nevertheless, additional studies are needed to fully elucidate the mechanism of BI-1 in plant thermotolerance.

## Methods

### Plant materials and gene cloning

The wheat cultivar Xiaobaimai and the *Arabidopsis* mutant *atbi1–2* (CS323793) and wild-type Columbia-0 (Col-0) were used in this study. *TaBI-1.1* (TRIAE_CS42_U_TGACv1_644608_AA2140670) was identified in a previous study [32]. The *TaFKBP62* (TRIAE_CS42_2DS_TGACv1_178636_AA0598780) coding sequence was amplified and inserted into the pLB vector (TIANGEN, China) (for primer sequences, see Additional file 7: Table S3). DNAMAN 6.0 software was used to determine amino acid sequence identity (Lynnon Biosoft, U.S.). Phylogenetic analyses were conducted using Molecular Evolutionary Genetics Analysis (MEGA 5.0, U.S.) software.

### Performance evaluation under heat treatment

The fusion vector *35S::TaBI-1.1*, in which the TaBI-1.1 coding sequence was cloned into the pCAMBIA1302 vector under the control of the CaMV 35S promoter, was described previously [32]. The *35S::TaBI-1.1* vector was transformed into *atbi1–2* to generate *35S::TaBI-1.1/ atbi1–2* transgenic line via the floral-dipping method. The *TaFKBP62* coding sequence was cloned into the pCAMBIA1302 vector under the control of the CaMV 35S promoter using an In-Fusion HD Cloning Kit (Clontech, United States) (for the primer sequences, see Additional file 7: Table S3). The *35S::TaFKBP62* fusion vector was transformed into Col-0 plants via the floral-dipping method. Homozygous T3 seeds were sterilized and germinated on MS medium [45] in a greenhouse at 22 °C under an 8-h light regime. For the phenotype analysis, 18-day-old plants were exposed to 45 °C for 6 h, and survival rates were measured after 7 days. Three biological replicates were performed.

For the ion leakage assay, 21-day-old plants were exposed to 45 °C for 3 h, and eight leaf discs (6 mm in diameter) were snipped from the leaves and placed in 5 mL of deionized water after heat treatment. After vacuuming for 30 min, the ion conductivity of the four genotypes was recorded as the initial conductivity, C1, and the ion conductivity of deionized water was recorded as C0. After incubating in boiling water for 30 min, we recorded the ion conductivity as C2, and the relative conductivity was calculated based on the formula:(C1-C0)/(C2-C0) × 100%. Ion conductivity was recorded using an FG3-B FiveGo™ conductivity meter (METTLER TOLEDO, Switzerland). Untreated plants were used as controls. Each treatment contained six independent replicates.

For hypocotyl-elongation assays, three-day-old plants grown in the dark were subjected to 45 °C for 2 h. After heat treatment, the plants were grown in the dark for three additional days, after which hypocotyl length was measured. The experiments included at least 36 seedlings for each genotype. Plants grown under normal conditions were used as controls.

### qRT-PCR analysis

Ten-day-old wheat seedlings grown at 22 °C were used for the heat treatments. For heat treatment, seedlings were exposed to 38 °C. For analyses of heat-responsive gene expression, eight-day-old Col-0, *atbi1–2,* and the *35S::TaBI-1.1/ atbi1–2* seedlings were exposed to 37 °C for 0 and 1 h. Three biological replicates were performed. The samples were harvested after treatment and were rapidly frozen in liquid nitrogen. Total RNA was extracted from the seedlings using the RNAprep pure Plant Kit (TIANGEN, China). qRT-PCR was performed on an ABI7500 system (ABI, U.S.). Primers used for qRT-PCR are listed in Additional file 7: Table S3.

### β-Glucuronidase (GUS) activity assay

The PBI::GUS fusion vector containing a 1.7 kb *TaBI-1.1* promoter, as well as the corresponding transgenic *Arabidopsis*, were obtained previously [32]. For heat treatment, five-day-old seedlings were exposed to 37 °C for 3 h. Untreated seedlings were used as controls. GUS staining was examined using a GUS histochemical assay kit (Real-Times, Beijing) according to the manufacturer’s instructions. Image analysis was performed using a Leica M165 FC stereomicroscope (Wetzlar, Germany).

### Yeast two-hybrid system

The BD-TaBI-1.1 and AD-TaBI-1.1 recombinant plasmids were obtained previously [32]. BD-TaBI-1.1 was used as a bait plasmid to screen a wheat cDNA library via yeast two-hybrid assays, which were performed according to the MATCHMAKER two-hybrid system user manual (Clontech, United States). The full-length TaFKBP62 coding sequence was amplified and cloned into pGBKT7 and pGADT7 to create BD-TaFKBP62 and AD-TaFKBP62, respectively. The TaFKBP62 fragments TaFKBP62-PPIase and TaFKBP62-TPR were cloned into pGADT7 to create AD-TaFKBP62-PPIase and AD-TaFKBP62-TPR recombinant plasmids (for the primer sequences, see Additional file 7: Table S3). The full-length coding sequences of AtBI-1 and ROF1 were amplified and cloned into pGBKT7 and pGADT7 to create BD-AtBI-1 and AD-ROF1, respectively. The TPR region of ROF1, ROF1-TPR, was cloned into pGADT7 to create the AD- ROF1-TPR recombinant plasmid (for the primer sequences, see Additional file 7: Table S3). To test the interactions, combinations BD-TaBI-1.1 + AD-TaFKBP62, AD-TaBI-1.1 + BD-TaFKBP62, AD-TaBI-1.1 + BD, BD-TaBI-1.1 + AD, AD + BD-TaFKBP62, BD + AD-TaFKBP62, AD + BD, AD-TaFKBP62-PPIase + BD-TaBI-1.1, AD-TaFKBP62-PPIase + BD, AD-TaFKBP62-TPR + BD-TaBI-1.1, AD-TaFKBP62-TPR + BD, BD-AtBI-1 + AD-ROF1, BD-AtBI-1 + AD, BD + AD-ROF1, BD-AtBI-1 + AD-ROF1-TPR, BD-AtBI-1 + AD, and BD + AD-ROF1-TPR were coexpressed in AH109 yeast cells and selected on SD/−Trp-Leu-His-Ade or SD/−Trp-Leu-His-Ade/X-α-gal medium for 5 days (Clontech, U.S.).

### Subcellular localization assay

The coding region of TaFKBP62 was amplified without a stop codon from wheat cDNA via PCR and inserted into the p16318GFP and pcDNA3.1-mRFP vectors (for the primer sequences, see Additional file 7: Table S3). The TaBI-1.1-GFP recombinant plasmid was obtained previously [32]. TaFKBP62-GFP and mRFP-HDEL, as well as TaBI-1.1-GFP and TaFKBP62-mRFP, were introduced into wheat protoplasts via polyethylene glycol-mediated transformation [46]. After incubation for 12 h in the dark at 22 °C, fluorescence signals were detected using a confocal laser-scanning microscope. For the subcellular localization of TaFKBP62 or TaBI-1.1 under heat treatment, TaFKBP62-GFP or TaBI-1.1-GFP was transformed into wheat protoplasts and the fluorescence signals were observed after treatment at 37 °C for 3 h.

### Bimolecular fluorescence complementation assay

The coding sequences of TaBI-1.1 and TaFKBP62 were amplified without stop codons via PCR using LA Taq DNA polymerase (Takara, Japan) and cloned into the medium vector pGWC-T according to the DNA Ligation Kit Ver.2.1 manual to generate pGWC-TaBI-1.1 and pGWC-TaFKBP62, respectively (for the primer sequences, see Additional file 7: Table S3). The sequences of TaBI-1.1 and TaFKBP62 from pGWC-TaBI-1.1 and pGWC-TaFKBP62 were further cloned into the destination vectors pEarlygate201-YN and pEarlygate202-YC, respectively, using gateway technology (Invitrogen, U.S.). The combined vectors were transformed into *Agrobacterium tumefaciens* strain GV3101 and introduced into *Nicotiana benthamiana* epidermal cells for transient expression as described previously [47]. YFP fluorescence was imaged using a confocal laser-scanning microscope after infiltration for 3 days.

### RNA-seq analysis

Eight-day-old wheat seedlings were treated at 40 °C and isolated at 1 h and 3 h. The samples were mixed for RNA-seq analysis of heat-treated wheat (Novogene, Beijing). Six-day-old Col-0 and *atbi1–2* plants were exposed to 37 °C for 1 h, and samples were collected for RNA-seq analysis of heat-treated *Arabidopsis* (Allwegene, Beijing). RNA-seq was performed using the HiSeq™2500 system (Illumina, U.S.).

## Additional files

Additional file 1: **Table S1.** Differentially expressed genes in the RNA-seq analysis of heat-treated wheat. (XLS 10704 kb)

Additional file 2: **Figure S1.** The number of BI-1 members in various plant species and phylogenetic analysis of the BI-1 family. (A) The number of BI-1 members in ten species. (B) Phylogenetic analysis of BI-1 family proteins. (TIF 1001 kb)

Additional file 3: **Figure S2.** AtBI-1 did not interact with ROF1 or the TPR region of ROF1. (A) The sequence diagrams of the full-length ROF1 sequence and the TPR domain of the ROF1. (B) The interaction between AtBI-1 and ROF1, as well as AtBI-1 and the TPR domain of the ROF1 by yeast two-hybrid analysis. (TIF 2385 kb)

Additional file 4: **Figure S3.** Subcellular localization of TaFKBP62 and TaBI-1.1 under heat stress. (TIF 6903 kb)

Additional file 5: **Table S2.** Differentially expressed genes between atbi1–2 and Col-0 in the heat-treated RNA-seq analysis. (XLSX 22 kb)

Additional file 6: **Figure S4.** Cluster analysis of the differentially expressed genes. (TIF 3543 kb)

Additional file 7: **Table S3.** Primer sequences used in this study. (XLSX 13 kb)

## Abbreviations

ABA: abscisic acid
ABRC: *Arabidopsis* Biological Resource Center
AD: pGADT7
BD: pGBKT7
BI-1: Bax inhibitor-1
BiFC: bimolecular fluorescence complementation
CaMV: cauliflower mosaic virus
ER: endoplasmic reticulum
FKBP: FK506-binding protein
FPKM: fragments per kilobase of transcript per million mapped reads
GO: Gene Ontology
GUS: β-glucuronidase
HSF: heat stress transcription factor
HSP: heat shock protein
KEGG: Kyoto Encyclopaedia of Genes and Genomes
PCD: programmed cell death
PPIase: peptidyl prolyl cis-trans isomerase
qRT-PCR: real time quantitative PCR
RNA-seq: RNA sequencing
SA: salicylic acid
TM: tunicamycin
TPR: tetratricopeptide repeat motif
YFP: yellow fluorescent protein
