# Supplementary figures and images for: Wheat Bax Inhibitor-1 interacts with TaFKBP62 and mediates response to heat stress
Source: BMC Plant Biol. 2018 Oct 26;18:259. doi: 10.1186/s12870-018-1485-0 (PMC6204060; doi:10.1186/s12870-018-1485-0)

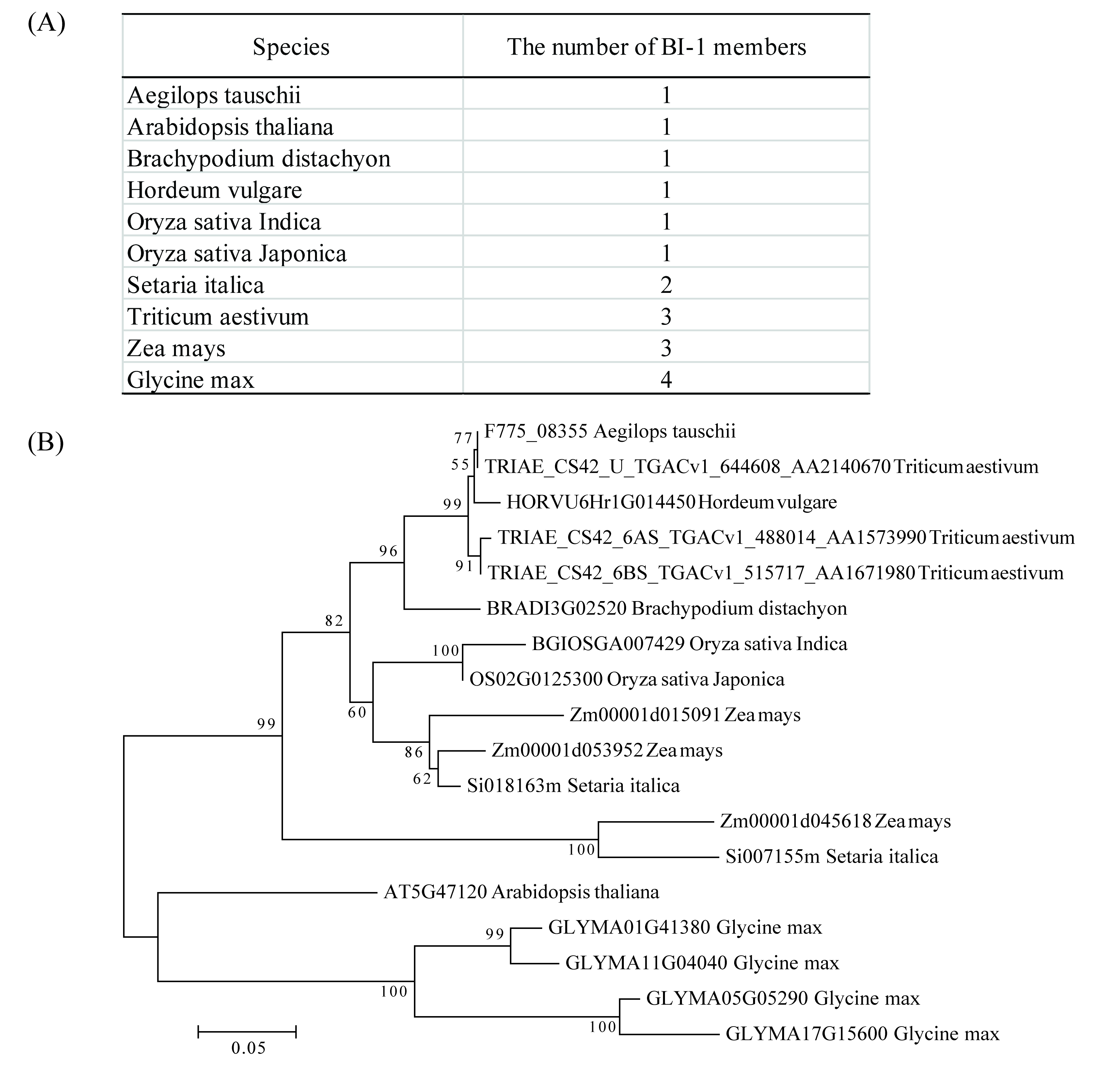

Supplement: Supplementary file 2 — Figure S1. The number of BI-1 members in various plant species and phylogenetic analysis of the BI-1 family. (A) The number of BI-1 members in ten species. (B) Phylogenetic analysis of BI-1 family proteins. (TIF 1001 kb) [file 12870_2018_1485_MOESM2_ESM.tif]

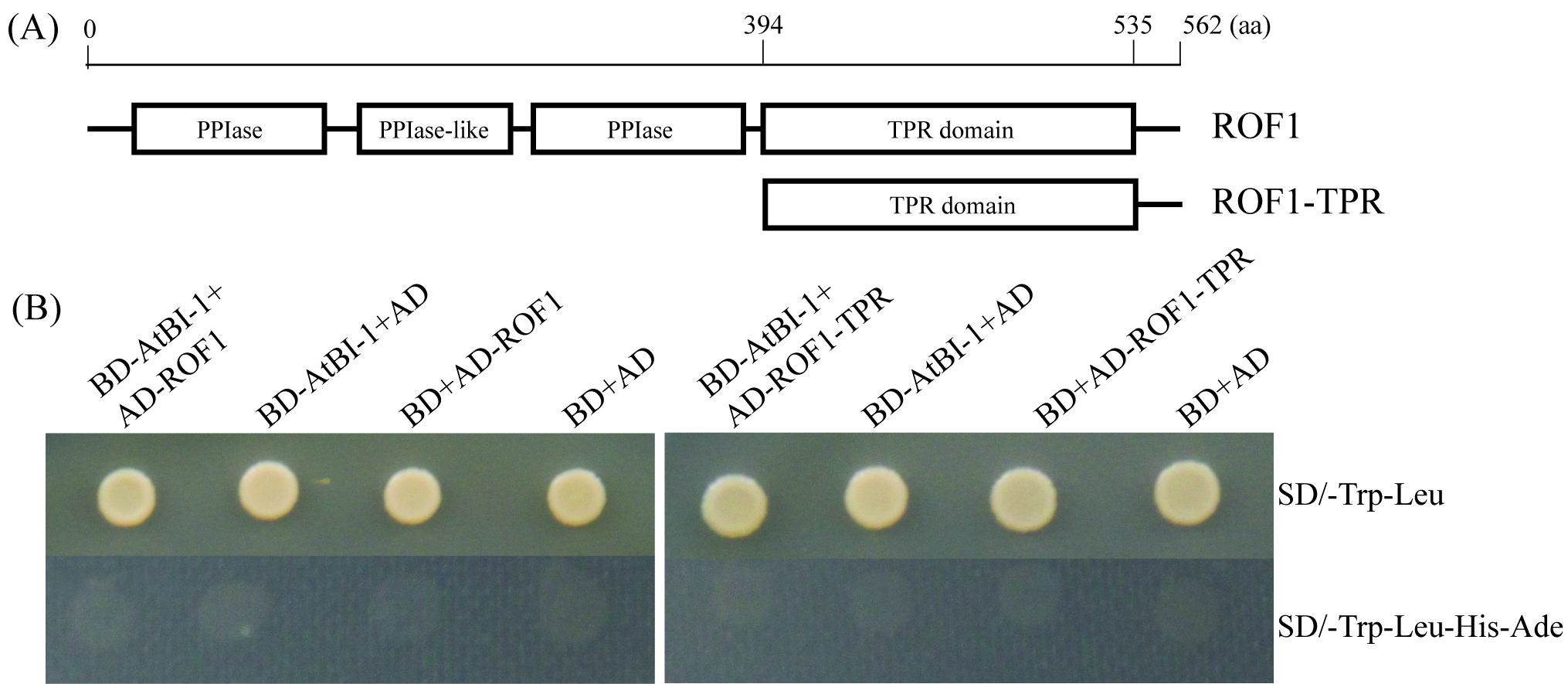

Supplement: Supplementary file 3 — Figure S2. AtBI-1 did not interact with ROF1 or the TPR region of ROF1. (A) The sequence diagrams of the full-length ROF1 sequence and the TPR domain of the ROF1. (B) The interaction between AtBI-1 and ROF1, as well as AtBI-1 and the TPR domain of the ROF1 by yeast two-hybrid analysis. (TIF 2385 kb) [file 12870_2018_1485_MOESM3_ESM.tif]

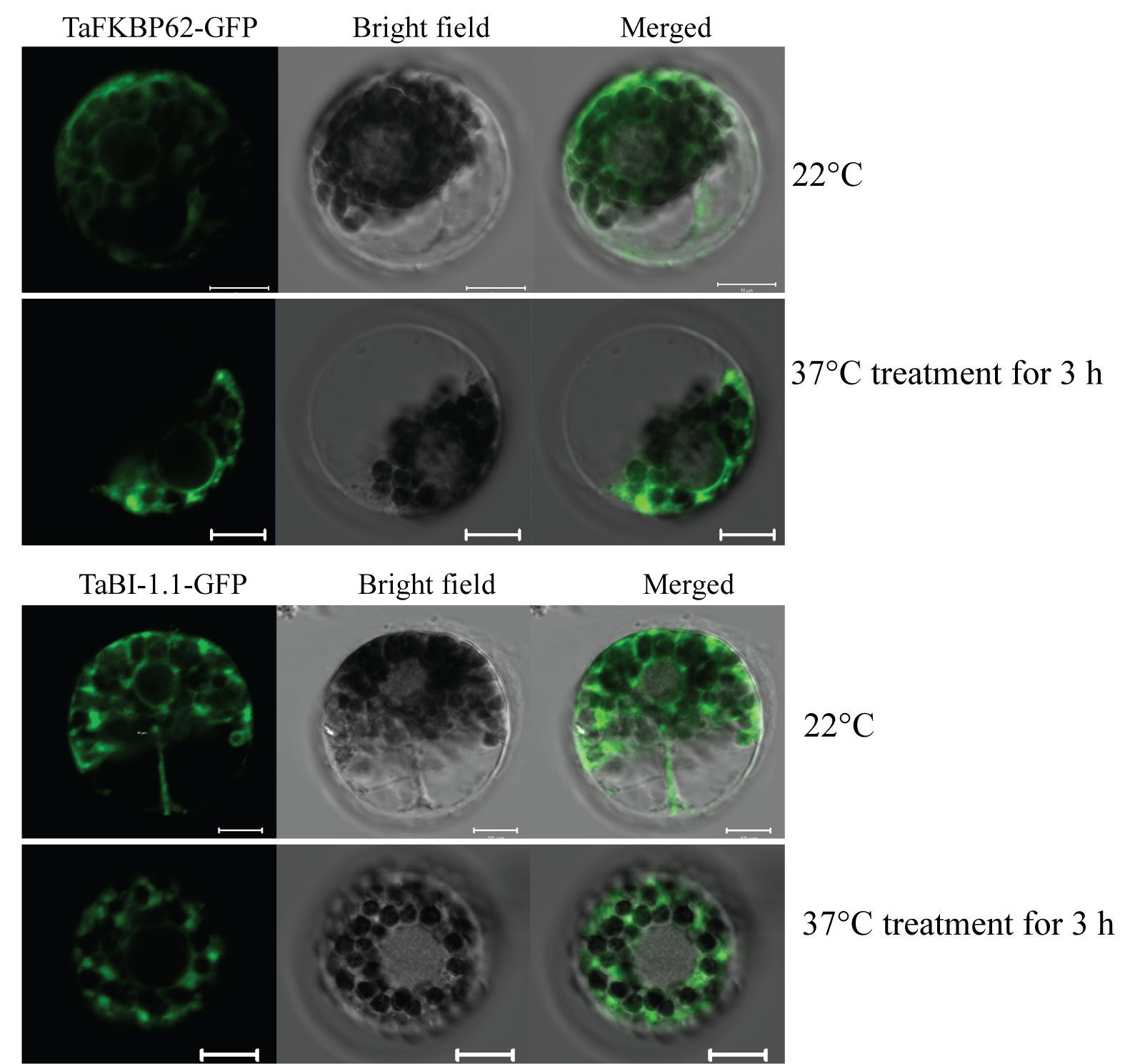

Supplement: Supplementary file 4 — Figure S3. Subcellular localization of TaFKBP62 and TaBI-1.1 under heat stress. (TIF 6903 kb) [file 12870_2018_1485_MOESM4_ESM.tif]
